# Effect of fermented dairy products on physicochemical and probiotic properties of tarhana in an in vitro gastrointestinal system

**DOI:** 10.1007/s13197-026-06593-z

**Published:** 2026-02-11

**Authors:** Merve İnce-Palamutoğlu, Recep Palamutoğlu, Betül Oruçoğlu, Cemal Kasnak, Salim Yılmaz

**Affiliations:** 1 Department of Nutrition and Dietetics, Faculty of Health Sciences, Afyonkarahisar Health Sciences University, 2078. Street, Dörtyol District, 03030 Afyonkarahisar, Turkey; 2 Department of Healthcare Management, Faculty of Health Sciences, Acıbadem Mehmet Ali Aydınlar University, 34752 Istanbul, Turkey

**Keywords:** Fermented dairy products, Tarhana, In vitro gastrointestinal system, Lactic acid bacteria

## Abstract

Tarhana is a fermented food that holds an important place in traditional Turkish cuisine. This study aimed to produce tarhana samples with increased probiotic content using various fermented dairy products, including yogurt, probiotic yogurt, and kefir. These samples were evaluated for their physicochemical, microbiological, antioxidant, and in vitro digestibility parameters. Significant differences were observed among the tarhana samples in terms of their physicochemical and microbiological characteristics (*p* < 0.05). The probiotic yogurt group exhibited the highest protein content, whereas kefir-based samples showed higher mineral content and a more neutral pH value. Although antioxidant activity and total phenolic content varied among groups, the highest values were obtained in the probiotic yogurt samples. During fermentation, a significant increase in *Lactobacillus* (*p* = 0.011) and *Lactococcus* (*p* = 0.006) counts was observed in the probiotic yogurt group, while no significant changes were detected in the other groups (*p* > 0.05). Throughout simulated digestion, differences in bacterial viability among groups were not statistically significant (*p* > 0.05), although the yogurt group maintained comparatively higher overall survival rates. The findings reveal that tarhana samples prepared using different fermented dairy products exhibited significant differences in terms of microbiological quality and nutritional content.

## Introduction

Fermented foods have been produced and consumed by humans since prehistoric times. Although their initial purpose was food preservation, they soon became valued for their distinctive flavors, textures, and nutritional benefits, and many have endured as part of cultural heritage across generations (Tamang et al. 2020; Sarıca and Özbay 2023). There are many traditional fermented foods produced worldwide, especially using grain-based raw materials (Madilo et al. 2024). One prominent example of these products in Türkiye is tarhana, which attracts attention with its nutritional value and cultural significance (Siddiqui et al. 2023).

According to standard number 2282 of the Turkish Standards Institute, tarhana is a semi-prepared food product obtained by kneading wheat flour, semolina, or a mixture of these, along with yogurt, vegetables, salt, and some spices, allowing them to ferment, then drying and grinding them (TSE 2004). Traditionally consumed as a soup during the winter months, tarhana presents a significant role in Turkish cuisine due to its fermented structure, rich nutritional content, and long shelf life (Koksel et al. 2024).

Among traditional fermented foods, dairy–cereal combinations are of particular interest because they combine the nutritional advantages of both food groups. Fermentation is known to enhance nutritional value and digestibility while extending shelf life. The production process of *tarhana* involves kneading wheat derivatives with yogurt and vegetables, followed by lactic acid fermentation lasting one to seven days. During fermentation, yeast species such as *Saccharomyces cerevisiae* and *Streptococcus thermophilus*, along with lactic acid bacteria including *Lactobacillus delbrueckii* subsp. *bulgaricus, Lactobacillus casei, and Lactobacillus acidophilus*, contribute to this process (Aydin et al. 2025).

Tarhana offers significant health benefits due to its high fiber content and its richness in vitamins B, calcium, iron, and zinc. It may also have positive effects on cholesterol levels, hypertension, and cardiovascular disease (Koksel et al. 2024). Epidemiological evidence indicates that higher yogurt consumption is associated with a lower risk of colorectal cancer, possibly due to the beneficial effects of live microorganisms present in fermented dairy products on the gut microbiota (Sun et al. 2022).

Tarhana prepared with fermented dairy products may acquire probiotic properties through the probiotic microorganisms involved in the fermentation process. Therefore, probiotic bacteria from fermented dairy products may be effective in helping to balance the human gut microbiota.

This study aimed to produce traditional tarhana enriched with probiotics by incorporating various fermented dairy products, including yogurt, probiotic yogurt, and kefir. Additionally, the survival and colon-reach potential of these probiotic microorganisms were assessed using an in vitro gastrointestinal system model.

## Materials and method

### Materials

Using five varieties of yogurt, kefir, and probiotic yogurt each of which were purchased from the market without name or brand, three distinct groups of traditional tarhana were produced for the study. Tarhana samples produced with yogurt were coded as A, B, C, D, and E; tarhana samples produced with probiotic yogurt were coded as F, G, H, I, and J; and tarhana samples produced with kefir were coded as K, L, M, N, and O. According to the label declarations of the yogurts used in tarhana samples produced with yogurt, the traditional starter cultures include *Lactobacillus delbrueckii* subsp. *bulgaricus* and *Streptococcus thermophilus*. For tarhana samples produced with probiotic yogurt, the product labels indicate that group F contains *Bifidobacterium animalis* subsp. *Lactis* and *Lactococcus lactis* subsp. *lactis*; groups G and H contain *Bifidobacterium* spp. and *Lactobacillus rhamnosus*; group I contain *B. animalis* subsp. *lactis*; and group J contains *Lactobacillus rhamnosus*. The kefir products used in tarhana production were labeled as containing at least 1 × 10⁶ cfu/g of viable probiotic microorganisms. In tarhana production, based on 100 g of flour, 40% yogurt/probiotic yogurt/kefir, 10% tomato paste, 5% dried onion, 2% red pepper powder, 1% salt, and 2.5% fresh yeast (*Streptococcus cerevisiae*) were added, and the mixture was homogenized in a laboratory mixer. The prepared tarhana was left to ferment in an incubator at 30 °C for 72 h. The tarhana dough was then spread as a thin sheet (3–5 mm) on a stainless-steel tray and dried in an oven at 55 °C for 48 h. Dried tarhana samples were ground in a laboratory mill and sieved through a 1 mm sieve before physicochemical analyses were performed (Erdoğan 2023). Eighteen grams of tarhana samples produced for the in vitro gastrointestinal system model was weighed and 200 cc of cold water was added, and the tarhana soup was cooked over low heat until it boiled. It was cooled to 70 °C and administered to the in vitro gastrointestinal system.

### Creation of in vitro gastrointestinal system model

A dynamic in vitro gastrointestinal system was established, comprising three compartments that represent the mouth, stomach, and small intestine. Transit times were set to 2 min (mouth), 2 h (stomach), and 2 h (small intestine) using a double-jacketed reaction vessel. To maintain a constant pH in the stomach and small intestine, real-time pH monitoring was performed, and 1 M NaOH or HCl was added as needed to adjust the pH.

**Fig. 1:**
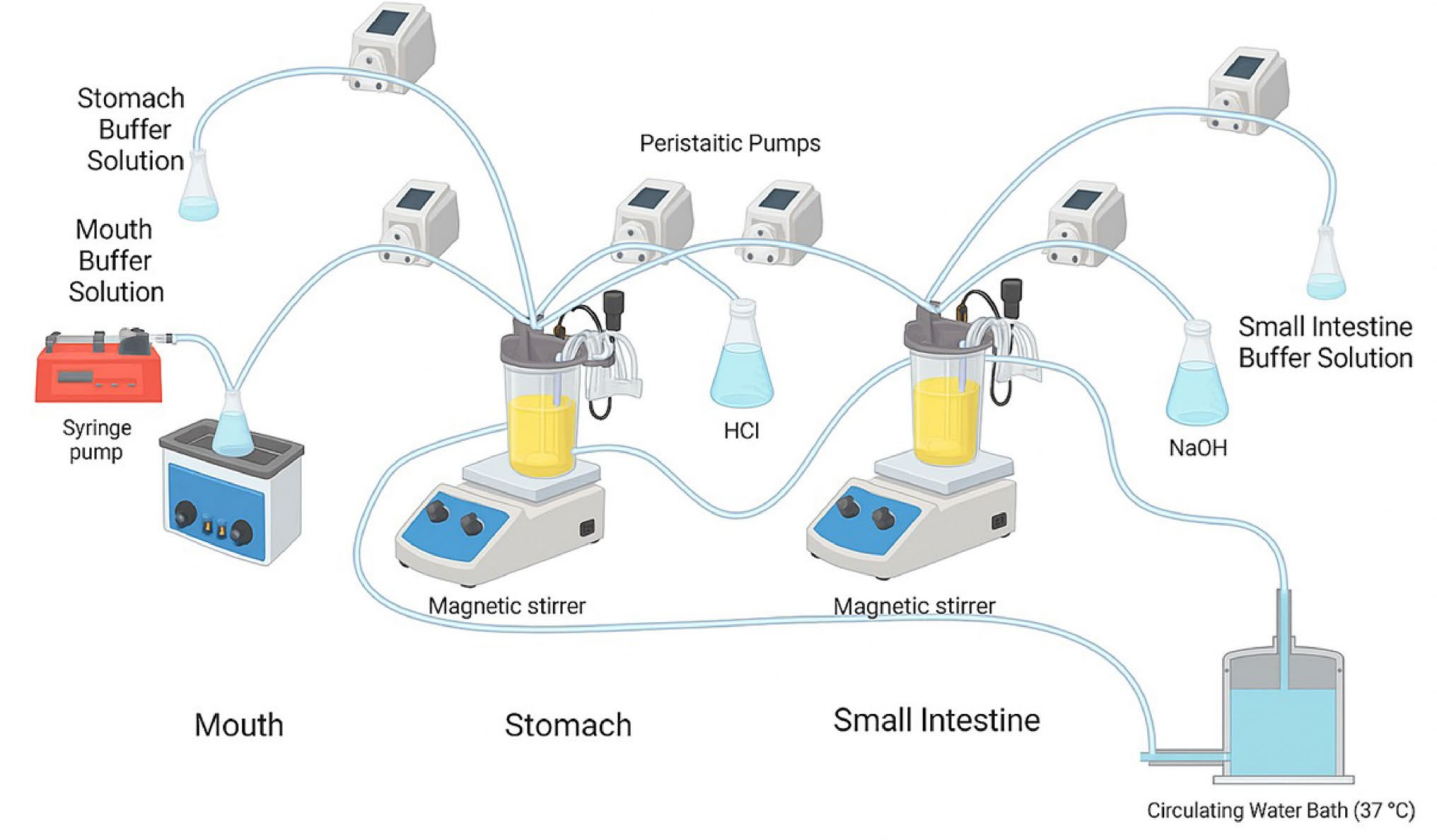
In vitro gastrointestinal system model

Simulated saliva contained 2 g/L α-amylase, 1 g/L mucin, 25 mL 0.3 M CaCl₂, and water to 1 L. It was added to the mouth compartment at a rate of 5 mL/min (0.05 mL/g sample). Stomach buffer contained 2.2 g/L KCl, 6.2 g/L NaCl, 1.2 g/L NaHCO₃, 0.22 g/L CaCl₂. Pepsin (3700 ppm) and mucin (23 g/L) were added to simulate gastric secretion, which was administered at 0.25 mL/min (0.05 mL/g). Upon transfer from the mouth to the stomach compartment, 0.2 M HCl was infused at 3.5 mL/min until pH reached 2.5 in 1 h, then maintained at that level for another hour with 0.9 mL/min HCl. Small intestine buffer composed of 0.6 g/L KCl, 5.0 g/L NaCl, and 0.25 g/L CaCl₂. To simulate intestinal secretion, 1 g/L pancreatin, 0.6 g/L lipase, and 12 g/L bile salts were added. This solution was added at a rate of 0.25 mL/min (0.05 mL/g). After transfer from the stomach, 1 M NaOH was added at a rate of 0.65 mL/min for 15–20 min to gradually raise the pH to 6.5, which was then maintained. All temperature settings, durations, and compositions of secretions and buffers were adapted from the in vitro gastrointestinal simulation protocol by Minekus et al. (Minekus et al. 2014; Çomak Göçer et al. 2016). The figure illustrating the in vitro gastrointestinal system used in the study is shown in Fig. 1.

Alfa-amylase, mucin, bile salt, pancreatin, and pepsin used in the study were obtained from Sigma-Aldrich (USA), CaCl_2_, NaCl, NaHCO_3_, and NaOH were obtained from AFG Bioscience (USA), KCl was obtained from TEKKİM (Turkey), and HCl was obtained from Honeywell (Germany). Ringer tablet and MRS Agar medium were obtained from Neogen Culture Media (Michigan, USA) and M17 Agar was obtained from Condalab Laboratorios (Madrid, Spain). Disposable sterile petri dishes were obtained from Fıratmed (Ankara, Turkey). Anaerobic conditioning reagent Anaerobentopf 2.5 l-Volumen, Microbiology Anaerocult A, and Anaerotest strips (Merck KGaA, Darmstadt, Germany) were used.

### Determination of crude protein content

The protein content of tarhana samples was determined using the Kjeldahl method. The nitrogen content of the samples was determined according to the AOAC (2000a, b, c, d). The total nitrogen value was multiplied by the protein factor (6.25) to calculate the percentage protein value (AOAC 2000a).

### Determination of crude fat content

A modified Soxhlet extraction method was used to determine the fat content of the samples. Approximately 5 g of sample was weighed into a cartridge, and extraction was performed using hexane for 150 min. The Soxhlet container was weighed and heated at 105 °C for 1 h in a drying oven, and the result was calculated as the percentage fat content (AOAC 2000b).

### Determination of moisture content

A digital precision balance was used to weigh 5 g of tarhana samples, which were then dried in an oven set at 105 °C until they reached a consistent weight. At the end of the process, the containers were cooled in a desiccator, and the dry matter content was calculated by precise weighing (AOAC 2000c).

### Determination of total ash content

The ash content of the tarhana samples was determined according to the AOAC (1997) method. Ten grams of tarhana samples were weighed into porcelain crucibles and placed in a muffle furnace at 550 °C until they turned into white ash. The results are given as % (w/v) (AOAC 1997).

### Determination of pH

Samples of tarhana weighing 10 g were collected and homogenized in 90 mL of distilled water using a homogenizer. The pH was determined with a pH meter.

### Determination of titration acidity

For titration acidity determination, 50 mL of 67% neutralized ethyl alcohol was added to 10 g of the tarhana samples, followed by filtration through filter paper (Whatman No.4, Cytiva). A few drops of phenolphthalein indicator solution were added and titrated with 0.1 N NaOH solution until a stable pink color was achieved. The result was expressed as lactic acid (%) based on the value consumed during the titration (AOAC 2000d).

### Color determination

Color determinations of tarhana samples were performed using an X-Rite Ci64x portable color spectrophotometer. The spectrophotometer quantifies the red, green, and blue components of each measurement and uses this data to determine the location of a color in color space using the L*, a*, b* metric (Schanda 2018).

### Total phenolic content analysis

Total phenolic content was determined in prepared tarhana soup samples and in 1 mL samples taken from the mouth at 2 min and from the stomach and small intestine at 0, 60, and 120 min after the in vitro gastrointestinal system model was administered, using the 2N Folin-Ciocalteu phenol reagent according to the method reported by Singleton and Rossi (1965). Results are expressed as gallic acid equivalents (GAE) (Singleton and Rossi 1965).

### Determination of DPPH radical scavenging capacity

Antioxidant capacity was determined using the DPPH (2,2-diphenyl-1-picrylhydrazyl) method, administered as 1 mL in the mouth at 2 min and in the stomach and small intestine at 0, 60, and 120 min after administration of soup samples from the prepared tarhana soup and the in vitro gastrointestinal system model, according to the method described by Brand-Williams et al. (1995) (Brand-Williams et al. 1995). The results were measured at 571 nm using a UV spectrophotometer (Şeker 2017).

### Microbiological analyses

Viable lactic acid bacteria count (log cfu/mL) in the prepared tarhana soup samples were determined at 0 min in the mouth immediately before administration to the in vitro gastrointestinal model. Tarhana soup samples were collected from the in vitro gastrointestinal model at 2 min in the mouth and at 0, 60, and 120 min in the stomach and small intestine. Viable microorganisms were counted. Before microbiological inoculations, serial dilutions of the appropriate decimals were prepared using 1⁄4 strength Ringer's solution under aseptic conditions. After preparing the serial dilutions, 1 ml of each dilution was transferred to sterile petri dishes, and the culture medium was added and incubated for 72 h. MRS Agar was used for the growth and enumeration of *Lactobacillus* cultures in the tarhana samples (Del Campo et al. 2005; Çomak Göçer 2016). M17 Agar was used for the growth and enumeration of *Lactococcus* cultures (Tabasco et al. 2007).

### Statistical analysis

The study was conducted with two replicates, and two samples from each replicate were analyzed in parallel. Statistical analysis of the data was performed using R 4.3.1 software. Due to the limited number of observations, robust or non-parametric analyses were used in the analyses. A mixed ANOVA model was applied for repeated measures to evaluate microbial and physicochemical changes over time and across groups. Post-hoc analysis was performed using Dunn's test, and significance levels were checked using the Bonferroni correction for multiple comparisons. Spearman correlation analysis was used to assess the relationship between physicochemical and microbial parameters. In the evaluations, a significance level of *p* < 0.05 was considered.

## Results and discussion

**Fig. 2:**
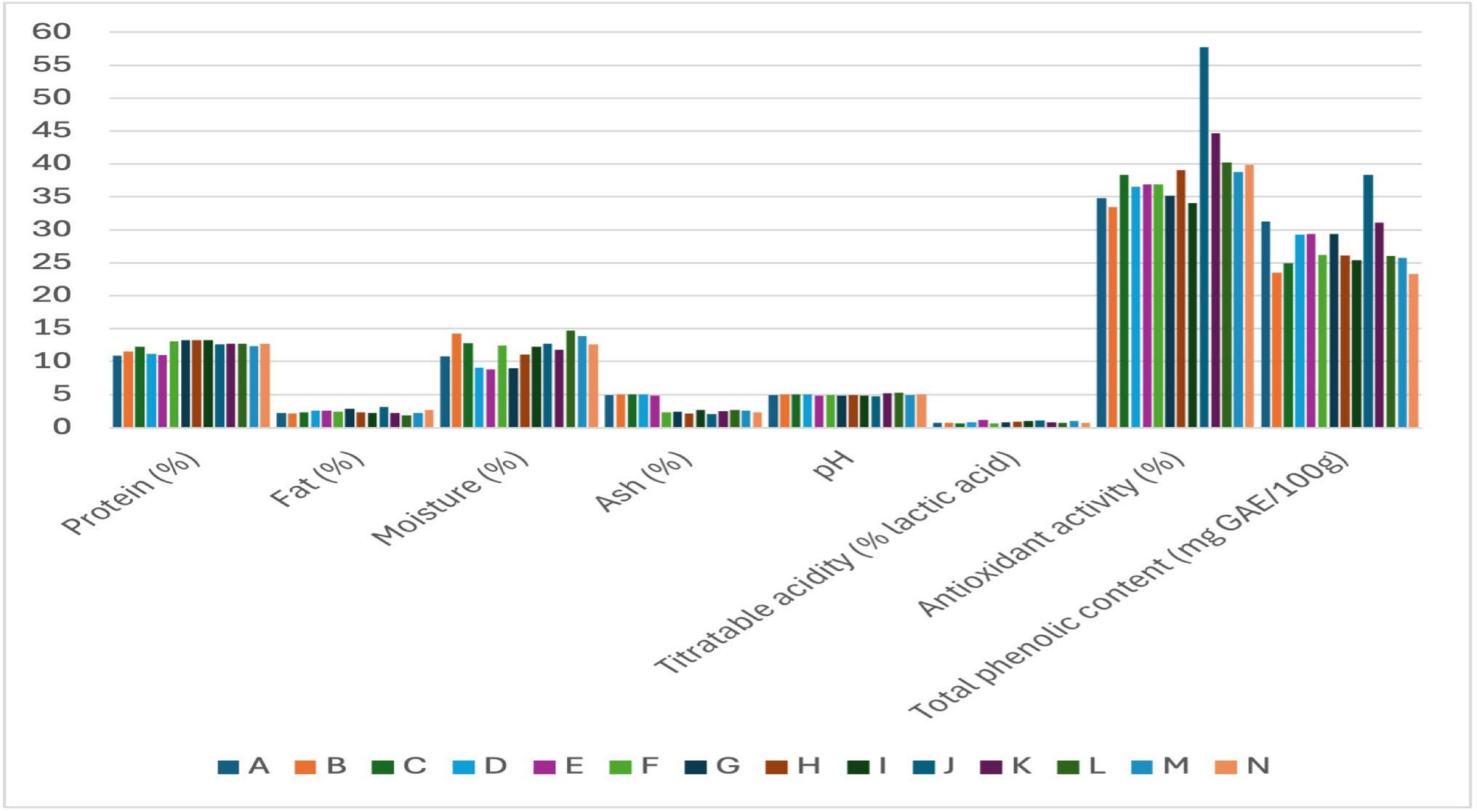
Comparison of analysis values in tarhana samples produced with various fermented dairy products

The main physicochemical properties of tarhana samples produced using various fermented dairy products are summarized in Table 1. While samples prepared with probiotic yogurt stood out in terms of protein content, significant differences were observed between groups in fat, moisture, and ash content. These differences are believed to be related to the composition of the dairy products used, their water-holding capacity, and the efficiency of the drying process. pH and titratable acidity values reflect the influence of fermentation conditions and microbial diversity; acidity levels vary depending on the microbial content of the tarhana. Groups prepared with kefir and probiotic yogurt had higher values for antioxidant activity and total phenolic content, and a positive correlation was observed between these two parameters. However, the higher antioxidant activity compared to phenolic content in some samples suggests that other antioxidant components contribute to the composition. Additionally, the bar chart comparing the analysis values of tarhana samples produced with different fermented dairy products is presented in Fig. 2. Color analysis revealed that products prepared with kefir exhibited lighter and yellow tones, while red tones were more dominant in yogurt-based samples. These findings suggest that the type of dairy product has a decisive effect on the chemical and visual properties of tarhana.

**Table 1:** Sorting tarhana samples prepared with different fermented dairy products based on analysis values

Order	Protein content (%)	Fat content (%)	Moisture content (%)	Ash content (%)	pH	Titratable acidity (% lactic acid)	Antioxidant activity (%)	Total phenolic content (mg GAE/100 g)	L*	a*	b*
1	G-13.24	J-3.13	L-14.68	I-2.68	L-5.31	E-1.22	J-57.76	J-38.33	M-78.47	B-10.94	J-26.43
2	I-13.21	G-2.87	B-14.28	L-2.68	K-5.17	J-1.08	K-44.65	A-31.24	I-77.38	J-10.91	A-24.45
3	H-13.21	N-2.65	M-13.92	M-2.58	O-5.12	I-0.99	O-42.15	K-31.06	E-76.76	N-10.62	B-24.44
4	F-13.08	O-2.59	C-12.79	K-2.49	N-5.05	M-0.99	L-40.21	E-29.38	A-76.34	K-10.46	K-24.15
5	N-12.70	E-2.57	J-12.73	O-2.39	D-5.00	O-0.95	N-39.91	G-29.38	O-76.16	F-10.45	H-23.78
6	K-12.69	D-2.52	N-12.61	G-2.34	B-5.00	H-0.90	G-39.05	D-29.29	H-75.96	A-10.40	N-23.54
7	L-12.67	F-2.34	F-12.39	F-2.31	C-4.97	G-0.86	C-38.30	O-27.52	G-75.86	L-10.20	C-23.36
8	J-12.61	C-2.28	I-12.27	N-2.28	F-4.95	K-0.86	E-36.85	F-26.19	D-75.43	C-9.88	F-23.30
9	O-12.60	H-2.26	K-11.78	A-2.24	H-4.93	D-0.81	M-36.76	H-26.10	C-73.96	H-9.86	G-23.18
10	M-12.30	I-2.22	H-11.11	E-2.13	A-4.89	B-0.76	D-36.51	L-26.01	L-73.74	G-9.77	E-23.16
11	C-12.29	A-2.21	A-10.82	H-2.12	M-4.88	A-0.72	F-35.20	M-25.75	J-73.52	E-9.72	D-23.13
12	B-11.53	M-2.20	O-10.40	C-2.03	I-4.82	L-0.72	A-34.82	I-25.39	K-73.38	D-9.67	O-23.09
13	D-11.16	K-2.19	D-9.10	D-2.01	G-4.82	N-0.72	H-34.09	C-24.95	N-73.37	O-9.61	I-22.84
14	E-10.99	B-2.14	G-9.03	J-2.01	E-4.80	C-0.68	B-33.42	B-23.44	B-73.15	M-9.26	L-22.71
15	A-10.93	L-1.82	E-8.81	B-1.89	J-4.74	F-0.63	I-33.29	N-23.26	F-71.25	I-9.15	M-22.06

L*: lightness, a*: red-green, b*:yellow-blue axes

In a comparative analysis of tarhana samples prepared using fermented milk products, the protein content was found to be significantly higher in the probiotic yogurt group (*p* = 0.0037). Furthermore, the ash content and pH values were statistically higher in the kefir group compared to the other groups (*p* < 0.05). However, no significant differences were found between the groups in terms of fat, moisture, acidity, antioxidant activity, total phenolic content, or color (*p* > 0.05) (Table 2).

**Table 2:** Comparison of tarhana samples prepared with different fermented dairy products based on physicochemical variables

Variable	Kefir^a^ Median (Q1–Q3)	Probiotic Yogurt^b^ Median (Q1–Q3)	Yogurt^c^ Median (Q1–Q3)	χ^2^	*p*	Post-hoc
Protein	12.67 (12.6–12.69)	13.21 (13.08–13.21)	11.16 (10.99–11.53)	11.180	0.004**	b > c
Fat	2.20 (2.19–2.59)	2.34 (2.26–2.87)	2.28 (2.21–2.52)	1.860	0.395	ns
Moisture	12.61 (11.78–13.92)	12.27 (11.11–12.39)	10.82 (9.10–12.79)	1.220	0.543	ns
Ash	2.49 (2.39–2.58)	2.31 (2.12–2.34)	2.03 (2.01–2.13)	6.860	0.032*	a > c
pH	5.12 (5.05–5.17)	4.82 (4.82–4.93)	4.97 (4.89–5.00)	6.540	0.038*	a > b
Acidity	0.86 (0.72–0.95)	0.90 (0.86–0.99)	0.76 (0.72–0.81)	0.728	0.695	ns
Antioxidant	40.21 (39.91–42.15)	35.20 (34.09–39.05)	36.51 (34.82–36.85)	4.460	0.108	ns
Total Phenolic	26.01 (25.75–27.52)	26.19 (26.10–29.38)	29.29 (24.95–29.38)	0.621	0.733	ns
L*	73.74 (73.38–76.16)	75.86 (73.52–75.96)	75.43 (73.96–76.34)	0.060	0.970	ns
a*	10.20 (9.61–10.46)	9.86 (9.77–10.45)	9.88 (9.72–10.40)	0.060	0.970	ns
b*	23.09 (22.71–23.54)	23.30 (23.18–23.78)	23.36 (23.16–24.44)	1.860	0.395	ns

χ^2^: Kruskal Wallis H test; N = 15; *p < 0.05; **p < 0.01, Q_1_: 25.percentile; Q_3:_ 75.percentile;

Different letters indicate statistically significant differences among groups (Dunn’s post-hoc with Bonferroni adjustment; *p* < 0.05); ns = not statistically significant (*p* > 0.05)

According to microbiological values, the counts of yeast, Lactobacillus, and *Lactococcus* differed between the groups at the beginning and end of fermentation. In particular, the J group reached the highest viability of both *Lactobacillus* and *Lactococcus* at the end of fermentation. These results demonstrate the impact of the fermented milk product used and the fermentation process on microbial populations (Table 3).

**Table 3:** Sorting tarhana samples prepared with different fermented dairy products based on yeast, *Lactobacillus*, and *Lactococcus* counts (log cfu/g) at the initial and end of fermentation

Order	Yeast count (log cfu/g, IOF)	Yeast count (log cfu/g, EOF)	*Lactobacillus* count (log cfu/g, IOF)	*Lactobacillus* count (log cfu/g, EOF)	*Lactococcus* count (log cfu/g, IOF)	*Lactococcus* count (log cfu/g, EOF)
1	M-8.76	A-5.68	M-8.92	J-9.10	L-8.97	J-9.41
2	D-8.48	I-5.65	O-8.37	N-8.81	M-8.64	N-9.21
3	E-8.21	G-5.63	D-8.34	M-8.70	D-8.46	K-8.76
4	F-8.06	E-5.62	E-8.28	K-8.66	E-8.14	D-8.60
5	H-7.97	B-5.62	L-7.82	L-8.57	O-8.06	H-8.43
6	L-7.94	N-5.62	A-7.8	C-8.39	A-7.98	I-8.35
7	A-7.91	L-5.58	B-7.72	H-8.20	B-7.86	C-8.34
8	C-7.70	C-5.57	C-7.61	F-7.97	C-7.72	M-8.33
9	J-7.59	F-5.55	N-7.60	G-7.96	N-7.72	L-8.33
10	B-7.58	O-5.54	H-7.59	D-7.91	G-7.68	G-8.20
11	G-7.54	M-5.53	G-7.55	B-7.89	H-7.60	A-8.07
12	N-7.35	K-5.52	F-7.53	E-7.86	F-7.49	E-8.01
13	I-7.28	D-5.48	K-7.23	A-7.81	I-7.13	F-7.90
14	O-7.21	H-5.47	J-7.18	I-7.77	K-7.13	O-7.79
15	K-7.20	J-5.36	I-7.02	O-7.74	J-7.11	B-7.45

IOF, initial of fermentation; EOF, end of fermentation

A significant decrease in yeast counts was observed in all groups during the fermentation process. Initial and final values decreased from 7.69 to 5.56 log cfu/g in the kefir group, from 7.69 to 5.53 log cfu/g in the probiotic yogurt group, and from 7.98 to 5.59 log cfu/g in the yogurt group, respectively (*p* < 0.001). A significant increase in *Lactobacillus* viability levels was observed only in the probiotic yogurt group (from 7.37 to 8.20 log cfu/g; *p* = 0.011), and this increase was not statistically significant in the kefir and yogurt groups (*p* = 0.092 and *p* = 0.934). Similarly, a substantial increase in *Lactococcus* viability was observed only in the probiotic yogurt group (from 7.40 to 8.46 log cfu/g; *p* = 0.006), while no significant change was observed in the other groups (kefir: *p* = 0.250; yogurt: *p* = 0.849). Furthermore, the repeated-measures ANOVA (Pillai’s trace) confirmed that the overall time effect on microbial counts was significant (*p* < 0.05), whereas the time × fermented dairy product interaction was not significant (*p* > 0.05), indicating that the fermentation effect followed a similar pattern across all groups (Table 4).

**Table 4:** Comparison of changes in microbial parameters in tarhana samples prepared with different fermented dairy products

Microbial parameter	Fermented dairy products	Initial (Mean ± SE)	End (Mean ± SE)	Within-group difference (initial-end)	Within-group *p*	Pillai’s *p*
Yeast (log cfu/g)	Kefir	7.69 ± 0.15	5.56 ± 0.15	2.13	< 0.0001***	0.6811
	Probiotic yogurt	7.69 ± 0.15	5.53 ± 0.15	2.16	< 0.0001***	
	Yogurt	7.98 ± 0.15	5.59 ± 0.15	2.38	< 0.0001***	
*Lactobacillus* (log cfu/g)	Kefir	7.99 ± 0.20	8.49 ± 0.20	− 0.51	0.0915	0.1608
	Probiotic yogurt	7.37 ± 0.20	8.20 ± 0.20	− 0.82	0.0113*	
	Yogurt	7.95 ± 0.20	7.97 ± 0.20	− 0.02	0.9340	
*Lactococcus* (log cfu/g)	Kefir	8.10 ± 0.22	8.48 ± 0.22	− 0.38	0.2499	0.1142
	Probiotic yogurt	7.40 ± 0.22	8.46 ± 0.22	− 1.06	0.0057**	
	Yogurt	8.03 ± 0.22	8.10 ± 0.22	− 0.06	0.8491	

Mixed repeated measurements ANOVA; *p < 0.05; **p < 0.01; ***p < 0.001; SE: Standard Error; Mixed repeated-measures ANOVA (within-subject factor: Time [Initial vs End]; between-subject factor: Fermented dairy product). Pillai’s trace values correspond to the overall effect of Time. Within-group *p*-values are derived from paired comparisons. **p < 0.001; *p < 0.01; *p* < 0.05; ns = not statistically significant (*p* > 0.05). Values are presented as mean ± standard error; units: log cfu/g

No significant difference was found in the viability levels of *Lactobacillus* and *Lactococcus* species between the groups at the mouth, stomach, and small intestinal stages (*p* > 0.05). While the change over time was not statistically significant in all three groups (Pillai's trace, *p* > 0.05), upon examining the mean values, it was observed that viability remained more stable throughout the digestive process, especially in the yogurt group (Table 5).

**Table 5:** In tarhana samples prepared with different fermented dairy products, changes in *Lactobacillus* and *Lactococcus* levels (log cfu/ml) across regions of the gastrointestinal system

GIS	Fermented dairy products	Initial (Mean ± SE)	End (Mean ± SE)	Within-group *p*	Pillai’s *p* (Time)
*Lactobacillus*					
Mouth	Kefir	2.30 ± 0.81	1.33 ± 0.88	0.128	0.130
Mouth	Probiotic yogurt	3.42 ± 0.98	3.34 ± 0.98	0.134	
Mouth	Yogurt	4.51 ± 0.64	4.64 ± 0.68	0.640	
Stomach	Kefir	0.82 ± 0.82	0.90 ± 0.91	0.780	0.203
Stomach	Probiotic yogurt	3.22 ± 0.93	3.18 ± 0.95	0.700	
Stomach	Yogurt	4.51 ± 0.66	4.68 ± 0.71	0.280	
Small Intestine	Kefir	0.50 ± 0.50	0.52 ± 0.51	0.860	0.694
Small Intestine	Probiotic yogurt	2.68 ± 1.09	2.76 ± 1.13	0.780	
Small Intestine	Yogurt	4.49 ± 0.58	4.45 ± 0.63	0.690	
*Lactococcus*					
Mouth	Kefir	3.66 ± 0.50	3.81 ± 0.46	0.533	0.995
Mouth	Probiotic yogurt	4.19 ± 0.55	4.17 ± 0.39	0.944	
Mouth	Yogurt	5.25 ± 0.25	5.12 ± 0.20	0.328	
Stomach	Kefir	4.02 ± 0.35	4.11 ± 0.14	0.785	0.133
Stomach	Probiotic yogurt	4.12 ± 0.42	4.24 ± 0.45	0.469	
Stomach	Yogurt	5.00 ± 0.38	5.76 ± 0.69	0.158	
Small Intestine	Kefir	3.83 ± 0.26	3.81 ± 0.44	0.950	0.133
Small Intestine	Probiotic yogurt	4.05 ± 0.43	3.99 ± 0.53	0.710	
Small Intestine	Yogurt	5.56 ± 0.35	5.02 ± 0.42	0.083	

GIS, gastrointestinal system

**Fig. 3:**
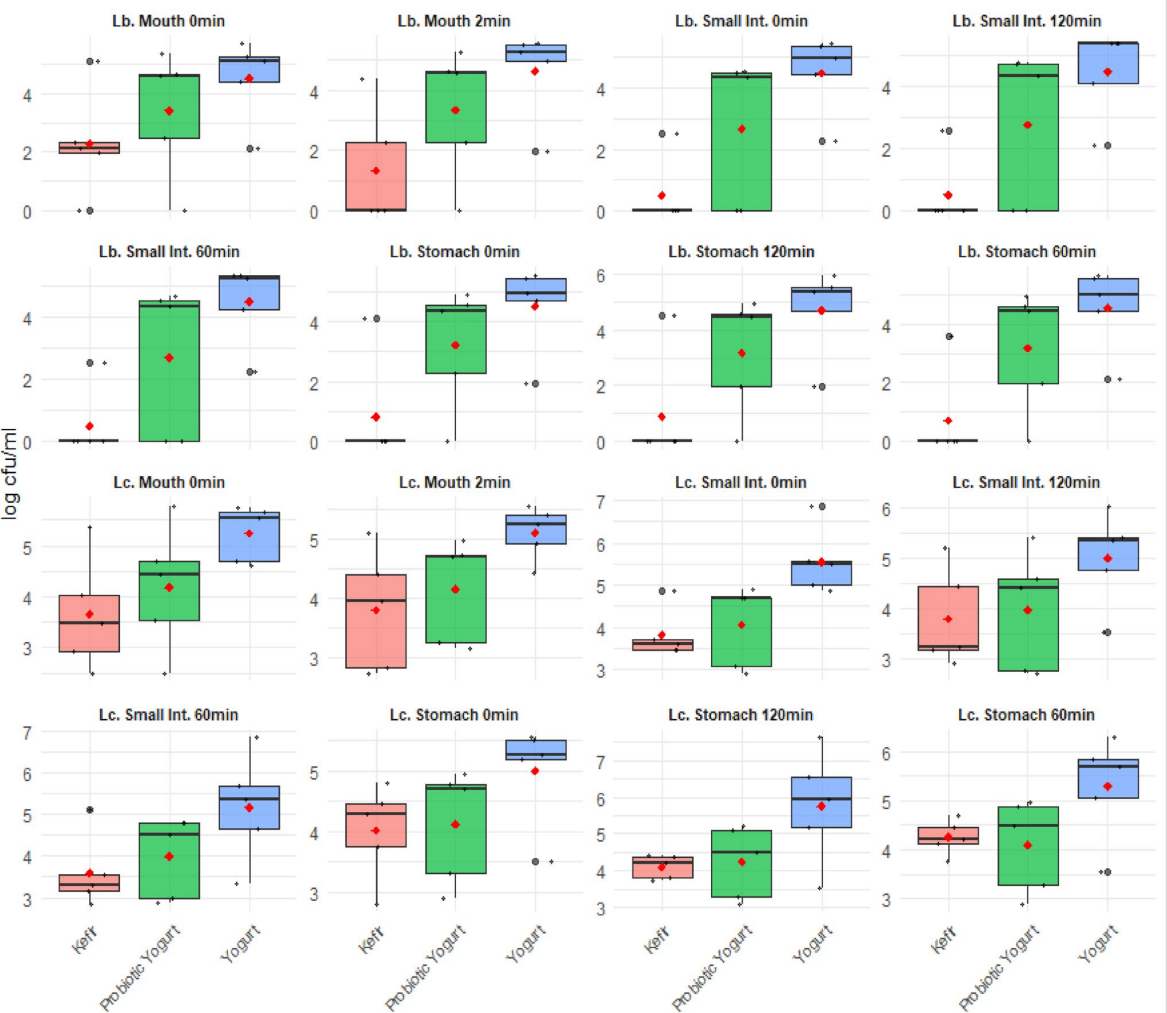
Distribution of probiotic viability tests in tarhana soups according to fermented dairy products and digestion stage values

Distribution of probiotic bacteria across simulated gastrointestinal phases is presented in Fig. 3, which illustrates *Lactobacillus* and *Lactococcus* viability patterns at different digestion stages (mouth, stomach, and small intestinal) for kefir-, probiotic yogurt-, and yogurt-based tarhana samples. The boxplots reveal considerable within-group variability, with the yogurt group generally maintaining higher median viability values compared with kefir and probiotic yogurt groups across most digestion stages. Despite these apparent differences, statistical analysis indicated no significant intergroup differences (*p* > 0.05). These data suggest that tarhana samples prepared with yogurt have the better potential to preserve probiotic viability throughout digestion.

**Fig. 4:**
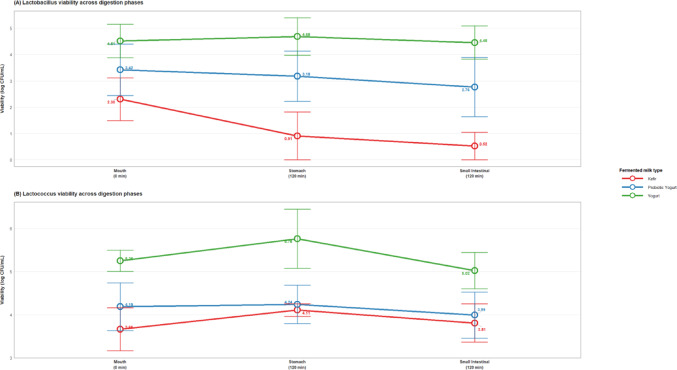
Probiotic viability across simulated gastrointestinal digestion phase. Note. Survival of **A** *Lactobacillus* and **B** *Lactococcus* strains across mouth, gastric, and intestinal phases in different fermented milk products. Error bars represent standard error of the mean (n = 3 per group)

Figure 4 further depicts mean probiotic viability trends throughout the digestion process. Viability of *Lactobacillus* species in the mouth phase started at 2.30 ± 1.81 log cfu/mL in the kefir group, 3.42 ± 2.19 log cfu/mL in the probiotic yogurt group, and 4.51 ± 1.42 log cfu/mL in the yogurt group (Fig. 4A). During stomach and small intestinal digestion, viability decreased markedly in the kefir and probiotic yogurt groups but remained elevated in the yogurt group (e.g., small intestinal phase at 120 min: kefir 0.52 ± 1.16; probiotic yogurt 2.76 ± 2.52; yogurt 4.45 ± 1.42 log cfu/mL). *Lactococcus* viability followed a more stable pattern across all fermented milk types (Fig. 4B), though the yogurt group consistently exhibited higher values during the stomach phase (120 min: yogurt 5.76 ± 1.53; kefir 4.11 ± 0.32; probiotic yogurt 4.24 ± 1.00 log cfu/mL). Both *Lactobacillus* and *Lactococcus* demonstrated distinct survival patterns across digestion phases, with yogurt-based tarhana showing superior maintenance of probiotic viability throughout the gastrointestinal system.

Ertan (2018), in his study in which yogurt was replaced with kefir at 25, 50, 75, and 100%, reported the protein content of tarhana samples as 14.44, 13.99, 13.69, and 13.51%, respectively, and as 14.62% in the control group. The lower protein content in tarhanas produced with kefir was explained by the fact that kefir contains less protein than yogurt (Ertan 2018). Arbağ (2022) determined the protein content in the range of 13.13–18.05% and reported the mean value as 16.76 ± 0.12% (Arbağ 2022). In this study, the protein content ranged from 10.93–13.24%, with the highest value being found in samples produced with probiotic yogurt and the lowest value being found in samples produced with yogurt. The observed differences in protein content are considered to result from variations in the composition of the starting materials (e.g., kefir, yogurt) rather than from microbial activity. While the findings are consistent with some studies, different results have been obtained due to differences in formulation. Furthermore, Oyewole and Ayo Odunfa (1989) observed an increase in protein content due to the increase in microbial protein during fermentation (Oyewole and Ayo Odunfa 1989). Fat content has been reported to vary between 0.66 and 6.26% by Arbağ (2022), whereas Şahin et al. (2025) found slightly higher and more consistent values ranging from 4.12 to 5.76% (Arbağ 2022; Şahin et al. 2025). In the study, the fat content was found to range from 1.82% to 3.13%, and no significant difference was observed between the groups. The wide ranges may be due to differences in the fat content of the dairy products used and the formulation. Although the TS 2282 Tarhana Standard does not specify a limit for fat content, the values obtained are consistent with those in the literature and traditional samples (TSE 2004).

Moisture content reported in the literature ranges from 6.43 to 10.29% (Ertan 2018; Arbağ 2022; Bardakçı and Karacabey 2024), whereas in the current study it was found between 8.81% and 14.68%, with no significant intergroup differences. Although the standard specifies a maximum moisture content of 10% (Soyyiğit 2004), moisture levels exceeded this value in some samples. This may be explained by the uneven distribution of the samples during drying and the fact that large pellets hinder moisture loss. It is well known that high moisture content can negatively impact quality, particularly in terms of oxidative and enzymatic degradation. Ash content reported in previous studies has shown considerable variation. Ertan (2018) found ash content to be 2.42% in the control group and between 2.41 and 2.43% in the kefir-substituted groups (Ertan 2018). Bardakçı and Karacabey (2024) reported higher values ranging from 7.53 to 7.79% (Bardakçı and Karacabey 2024), while Arbağ (2022) recorded values between 1.25 and 1.96%, with a mean of 1.72 ± 0.16% (Arbağ 2022). In the present study, ash content ranged from 2.03 to 2.49%, with statistically significant differences observed between the groups. The increased ash content in kefir-based samples is likely due to the high mineral content of kefir.

Regarding pH values, Ertan (2018) found values of 4.75–4.99 at the initial of fermentation and 4.31–4.45 at the end of the fifth day, with the lowest pH values reported in the kefir-produced samples (Ertan 2018). Bardakçı and Karacabey (2024) found pH values in the range of 4.75—4.86, while Arbağ (2022) found pH values in the range of 4.46—4.84 (Arbağ 2022; Bardakçı and Karacabey 2024). In this study, the sample produced with kefir had the highest pH value at 5.12, while the sample made with probiotic yogurt had the lowest pH value at 4.82. The difference between the groups was not statistically significant. The higher pH value of kefir, despite its lower acidity, may be due to its buffered, weakly acidic structure.

Ertan (2018) observed that titratable acidity rose from 1.50% in yogurt-containing samples to 1.70% with increasing kefir substitution. Notably, kefir was found to significantly enhance acidity at substitution rates of 50% or higher (Ertan 2018). Dağ (2019) stated that the effect of different yogurt culture concentrations on pH and acidity was not statistically significant (*p* > 0.05) (Dağ 2019). Arbağ (2022) determined the mean titratable acidity to be 1.60 ± 0.07% and reported substantial effects of yogurt type and substitution rate (*p* < 0.01), while interactions were insignificant (Arbağ 2022). In this study, the sample prepared with probiotic yogurt had the highest acidity values at 0.90%, while the sample with kefir had lower acidity values at 0.86% and 0.76%, respectively. The higher total acidity in kefir samples, despite the high pH, indicates the buffering effect of weak acids. Consequently, the type of dairy product used significantly affected the physicochemical properties of tarhana, such as protein, fat, moisture, ash, pH, and titratable acidity. The findings are broadly consistent with the existing literature, although some observed differences are attributed to variations in formulation, type of dairy product, and fermentation and drying conditions. Similarly, Demirci et al. (2018) reported that replacing yogurt with kefir in tarhana increased fermentation activity, reflected by higher acidity and a faster pH decrease. They attributed this to the synergistic action of lactic acid bacteria and yeasts in kefir. Consistent with these findings, the present study confirms that the type of fermented dairy product affects fermentation dynamics and acidity development in tarhana (Demirci et al. 2018).

Bardakçı and Karacabey (2024) reported total phenolic content (TPC) and total antioxidant activity (TAA) values in tarhana samples dried with Refractance Window™ (RWM) technology as 35.03–42.19 mg GAE/100 g (db) and 16.67–20.75 mg TE/100 g (db), respectively (Bardakçı and Karacabey 2024). It has been stated that the drying method and formulation affect the release and bioaccessibility of these compounds. In this study, the highest total phenolic content was found in the sample produced with yogurt, at 29.29 mg GAE/100 g. Lower TPC values were obtained in samples prepared with probiotic yogurt (26.19) and kefir (26.01). Although no statistically significant difference was found between the groups, this difference is thought to be due to the potential of microbial cultures to modify or metabolize phenolic compounds. This suggests that the number of phenolic compounds is affected not only by the formulation but also by fermentative microbial activity. Arbağ (2022) determined the total phenolic content in tarhana samples to be between 3.71 and 17.34 g GAE/kg, with a mean of 10.33 ± 0.02 g GAE/kg. In the analysis of variance, yogurt type, substitution rate, and interaction factors were all found to be statistically significant in terms of TPC (*p* < 0.01) (Arbağ 2022). In the same study, antioxidant activity values ranged from 24.26% to 63.15%, with a mean of 30.30 ± 0.25%. Similarly, all factors were found to have a statistically significant effect on antioxidant capacity (*p* < 0.01). These findings are consistent with the results of this study and support the impact of the dairy product used on the phenolic structure and antioxidant capacity. These findings indicate that phenolic compounds are affected not only by the formulation but also by fermentative microbial activity. When the color properties of tarhana samples were examined, differences in L*, a*, and b* values were observed depending on the type of dairy product. In this study, the highest L* value was measured in the probiotic yogurt sample (75.86), and the lowest in the kefir sample (73.74). Redness values ranged from 9.86 to 10.20, and b* values ranged from 23.09 to 23.36. Although there were arithmetic differences in all parameters, no statistically significant differences were found (*p* > 0.05). Ertan (2018) reported L* values between 68.32 and 69.20, a* values between 8.66 and 9.71, and b* values between 26.11 and 26.66 in samples prepared with kefir substitution and stated that these differences were statistically insignificant (Ertan 2018). Arbağ (2022) reported that yogurt type, substitution rate, and all interactions had significant effects on L*, a*, and b* values (*p* < 0.01), and the mean L*, a*, and b* values were 73.08 ± 0.15, 11.02 ± 0.09, and 27.01 ± 0.10, respectively (Arbağ 2022). The results obtained in this study are generally consistent with the existing literature, and it is considered that the differences in color parameters may be attributed to microbial activity, the pigment content of dairy products, and the drying conditions.

Işık Doğan and Yılmaz (2023) evaluated the change in *Lactobacillus* count during fermentation in traditional tarhana samples from different regions. While the *Lactobacillus* count in Isparta tarhana was initially 9.082 log cfu/g, it decreased to 8.591 log cfu/g at 24 h. In Maraş tarhana, this value decreased from 7.499 to 7.112 log cfu/g (*p* < 0.05). On the other hand, an increase was observed from 7.789 log cfu/g to 8.093 log cfu/g in Bursa tarhana. Similarly, in Kastamonu tarhana, the initial value of 6.990 log cfu/g increased to 7.180 log cfu/g at 24 h; however, the difference between the 5th and 7th days was not found to be statistically significant (*p* > 0.05). In Uşak tarhana, a rapid increase in the *Lactobacillus* count occurred; The initial value, which was 6.524 log cfu/g, reached 8.854 on day 1 and 9.101 on day 3 (Işık Doğan and Yılmaz 2023).

Results regarding *Lactococcus* and yeast counts in traditional tarhana samples from different provinces reveal the impact of the fermentation process on microbial dynamics. Işık Doğan and Yılmaz (2023) reported that the number of *Lactococcus* species increased significantly during fermentation in Isparta and Kastamonu samples; however, they noted that the increase was more limited or not statistically significant in Maraş, Bursa, and Uşak samples. Fluctuations were observed over time, particularly in the Uşak samples, and counts could not be obtained on day 21 due to yeast dominance (Işık Doğan and Yılmaz 2023). These differences are thought to be due to changes in the initial microbial load, the type of culture used, and the formulation.

A similar change was observed in terms of yeast population. Işık Doğan and Yılmaz (2023) reported a significant increase in yeast counts in Isparta tarhana after 24 h of fermentation. Soyyiğit (2004) reported changes in yeast and mold levels over time in samples from Isparta, Maraş, Bursa, and Uşak, and noted a significant decrease in yeast counts in some samples as fermentation progressed. This is attributed to increased acidity and the dominance of LAB, which limits yeast growth (Işık Doğan and Yılmaz 2023; Soyyiğit 2004). In the study, a significant increase in *Lactobacillus* levels was observed in tarhana samples prepared with probiotic yogurt. *Lactococcus* counts increased slightly in the yogurt group and more in the kefir group, but these differences were not statistically significant. The increase in the probiotic yogurt group is likely attributable to the dominance of species such as *Streptococcus thermophilus* and *Lactococcus lactis* in the medium. In contrast, in samples prepared with kefir, some heterofermentative LAB species are thought to limit this increase. Generally, the type of dairy product used plays a significant role in the microbial profile of tarhana and the progression of the fermentation process.

The findings of this study are consistent with previous reports, which emphasize that the type of fermented dairy product affects the physicochemical and microbiological properties of tarhana. Similar to Demirci et al. (2018), kefir-based formulations showed higher fermentation activity due to the synergistic interaction between lactic acid bacteria and yeasts. The greater microbial diversity in kefir and probiotic yogurt may have promoted proteolytic activity, enhanced protein and phenolic compound contents and contributing to improved nutritional and functional quality (Demirci et al. 2018).

Ince Palamutoğlu et al. (2023) evaluated the essential components, antioxidant activities, and LAB levels of kefir samples in the in vitro digestive system. For this purpose, a total of 12 kefir samples were used, comprising five produced using home-based methods, two using starter cultures, and five using industrial methods. *Lactobacillus* and *Lactococcus* levels were monitored using a three-stage in vitro digestion model (mouth, stomach, small intestine). In the study, a decrease in LAB viability was observed throughout the digestive process in all kefir groups; statistically significant differences were found between the groups, particularly during the stomach phase (*p* < 0.05) (İnce Palamutoğlu et al. 2023). In tarhana samples, *Lactobacillus* counts varied throughout digestion in kefir, probiotic yogurt, and yogurt-prepared groups. The counts, which began in the mouth phase, decreased significantly in the kefir group, whereas they remained more stable in the yogurt group. Similar trends were observed in the stomach and small intestine phases; however, the differences between the groups were not statistically significant (*p* > 0.05). *Lactococcus* levels increased in the mouth and stomach phases and decreased in the small intestine. However, these changes were not significant. The increase in *Lactococcus* in the probiotic yogurt group was attributed to the dominance of *Streptococcus thermophilus* and *Lactococcus lactis*. At the same time, the potential suppressive effect of heterofermentative LAB species was emphasized in the kefir group.

Although conducting the study using an in vitro gastrointestinal system represents an innovative approach, this study has certain limitations. First, bacterial identification was restricted to genus-level enumeration, and molecular confirmation (e.g., 16S rRNA sequencing) could not be performed to verify the species-level composition. Second, due to resource constraints, each analysis was conducted in two replicates with two parallels, which may limit the statistical power of the findings. Finally, because of the difficulty in obtaining ethical approval for sensory analyses in our country, sensory evaluation was not included in the study design; therefore, potential changes in the organoleptic properties of tarhana produced with different fermented dairy products could not be assessed. Future studies should include molecular characterization, larger sample sizes, and sensory analysis to provide a more comprehensive understanding of the functional and consumer aspects of probiotic-enriched tarhana.

## Conclusion

This study demonstrated that the type of fermented dairy product (yogurt, probiotic yogurt, or kefir) used in tarhana production significantly influences its physicochemical and probiotic properties. Among the formulations, tarhana produced with probiotic yogurt exhibited the highest protein content and supported the greatest increases in *Lactobacillus* and *Lactococcus* viability during fermentation, indicating its potential to enhance tarhana’s functional quality by maintaining probiotic survival under simulated gastrointestinal conditions. No statistically significant change in viability was observed for these species in the yogurt and kefir groups. In contrast, kefir-based tarhana showed higher mineral content and buffering capacity, suggesting a different but complementary functional contribution.

The observed variations in probiotic survival among tarhana samples can be attributed to physicochemical properties, including pH, titratable acidity, and matrix viscosity. The more neutral pH and higher buffering capacity of kefir may reduce acid-induced stress. In contrast, the denser protein–polysaccharide structure and higher fat content in yogurt and probiotic yogurt create a protective matrix that limits acid and bile salt diffusion during digestion. These factors likely contribute to the superior maintenance of bacterial viability observed in yogurt-based tarhana. Overall, the interplay between acidity, buffering capacity, and matrix composition plays a decisive role in sustaining probiotic functionality throughout fermentation and gastrointestinal transit.

These findings highlight that using probiotic yogurt as a fermentation matrix can improve both the nutritional and microbial functionality of traditional tarhana, supporting its development as a probiotic-rich functional food. Further research incorporating molecular identification and sensory evaluation will strengthen the understanding of its technological and consumer potential.

## Data Availability

The data obtained in this study are available from the corresponding author. upon request.
